# Market power and food loss at the producer-retailer interface of fruit and vegetable supply chains in Germany

**DOI:** 10.1007/s11625-021-01083-x

**Published:** 2022-01-15

**Authors:** Ronja Herzberg, Thomas Schmidt, Markus Keck

**Affiliations:** 1 Thünen Institute of Market Analysis, 38116 Braunschweig, Germany; 2 Chair for Urban Climate Resilience, Center for Climate Resilience, University of Augsburg, 86156 Augsburg, Germany

**Keywords:** Food loss and waste, Agriculture, Horticulture, Retail, Sociology of markets, Primary production

## Abstract

Food loss and waste are associated with an unnecessary consumption of natural resources and avoidable greenhouse gas emissions. The United Nations have thus set the reduction of food loss and waste on the political agenda by means of the Sustainable Development Goal Target 12.3. The German Federal Government committed itself to this goal by implementing the National Strategy for Food Waste Reduction in 2019. However, this policy approach relies heavily on voluntary action by involved actors and neglects the possible role of power imbalances along the food supply chain. While current research on food loss and waste in industrialised countries predominantly focuses on the consumer level, this study puts emphasis on the under-researched early stages of the food supply chain from the field to retailers’ warehouses. Based on 22 expert interviews with producers, producer organisations and retailers, this article identifies major inter-stage drivers of food loss in the supply chains for fresh fruit and vegetables in Germany. Its main novelty is to demonstrate how market power imbalances and risk shifting between powerful and subordinate actors can reinforce the tendency of food loss on the part of producers further up the supply chain. Results indicate that prevalent institutional settings, such as contractual terms and conditions, trading practices, ordering processes, product specifications, and communication privilege retailers and encourage food loss. The mechanisms in which these imbalances manifest, go beyond the European Commission’s current legislation on Unfair Trading Practices. This study suggests a research agenda that might help to formulate adjusted policy instruments for re-structuring the German fruit and vegetable markets so that less food is wasted.

**Supplementary Information:**

The online version contains supplementary material available at 10.1007/s11625-021-01083-x.

## Introduction

Reducing food loss is a global challenge to create more sustainable agri-food systems (Keck 2021): worldwide one third of food is wasted (Gustavsson et al. 2011) representing 4.6 billion metric tonnes in annual carbon dioxide emissions or 9% of global greenhouse gas emissions (Poore and Nemecek 2018). A total of about twelve million tonnes fresh mass was wasted in Germany in 2015 (Schmidt et al. 2019). A political framework to reduce food loss and waste is given by the United Nations, the EU and national regulations: The Sustainable Development Goal (SDG) Target 12.3, the waste directive and its delegated acts regarding food loss and waste at EU level (European Commission 2019; European Parliament 2018), supplemented by the National Strategy for Food Waste Reduction (BMEL 2019a). Within this political framework, food loss prior to retail is addressed less ambitiously (Parfitt et al. 2021; Porter et al. 2018; Soma et al. 2021; Stenmarck et al. 2016). In particular, pre-harvest and harvest loss is not even accounted for within the EU monitoring guidelines (European Parliament 2002) and the SDG 12.3 does not strive for a defined reduction target for supply chain stages prior to retail (Flanagan et al. 2019). Similarly, in research this part of the value chain is often neglected as opposed to consumption stages (Herzberg et al. 2020), although it is also associated with resource use and climate-relevant emissions (Porter et al. 2018; Spang et al. 2019).

As in Germany 30% of the overall food loss and waste occurs in primary production and processing (Schmidt et al. 2019) and loss rates prior to harvest are still unknown, this part of the food supply chain deserves further attention by the scientific community. The paper examines drivers of food loss in the early food supply chain at the example of fresh fruit and vegetables in Germany. Although fruit and vegetable production plays a minor role in Germany with a yield of almost five million tonnes per year (BMEL 2019b), food loss of fruit and vegetables in primary production from harvest onwards accounts for 21% of the entire food loss volume in the country (Schmidt et al. 2019).

There have been various studies on the drivers of fruit and vegetable losses both, internationally and in Germany (Baker et al. 2019; Beausang et al. 2017; Gillman et al. 2019; Hooge et al. 2018; Johnson et al. 2019). However, only very few studies deal with the underlying relationship and power constellations between supply chain actors as potential drivers of food loss on other supply chain stages. If they do so, they focus on different product categories or geographic regions (Devin and Richards 2018; Ghosh and Eriksson 2019; Soma et al. 2021).

The relationship and interactions between supply chain actors as well as the underlying power constellations can however be crucial, as food loss often comes along with economic risk and loss. It has been stated that food loss can in many cases be reduced to a minimum for economic considerations (FAO 2019; Koester 2014). However, there is a lacking incentive for buyers to optimise activities if economic decisions result in food loss and accompanying costs shouldered by upstream supply chain actors (Cattaneo et al. 2020). To approach the depicted research gap, this paper combines an analysis of interactions between different supply chain stages and actors on the one hand and its potential facilitation of food loss in the upstream supply chain, on the other hand. In this context, power constellations need to be considered, since it has been shown that the food system is increasingly dominated by large actors, in the case of horticulture particularly on the retailing side (Bundeskartellamt 2014; Wiggerthale 2021). Piras et al. (2018), Feedback (2017) and Eriksson et al. (2017) argue for other countries that Unfair Trading Practices resulting from power imbalances can generate food loss and waste. In the face of a highly competitive market situation and rising consumer claims (Hooge et al. 2017; Loebnitz et al. 2015), retailers can use their superior market position to set standards and terms and conditions, determine business habits and contractual terms, and delegate economic risks and costs onto suppliers (Devin and Richards 2018; Eriksson et al. 2017; Skorbiansky and Ellison 2019).

The European Commission is already paying attention to the topic of market power imbalances and Unfair Trading Practices (UTPs) in agricultural supply chains by issuing a directive to protect suppliers of agricultural produce as defined by their annual sales (European Parliament 2019). The present study discusses whether market power imbalances, trading practices, and the related bearing of risks and costs between supply chain actors have an effect on the occurrence of food loss in the upstream supply chain. To fill the depicted research gap, the paper answers the following questions:

1. Through which mechanisms become structural or market power imbalances apparent in fruit and vegetable supply chains in Germany?
2. How do interactions, shaped by power imbalances, result in food loss?
3. At which stages of the supply chain does this loss occur?

Throughout the paper we use the term “food loss” for losses prior to the retail stage, including harvest and pre-harvest losses, as applied by the Food and Agriculture Organization of the United Nations (FAO 2019). “Food waste”, on the other hand, only occurs at the retail and consumer level.

On the next pages, we embed our research questions into the current debate on circular economies and present a theoretical framework informed by the sociology of markets. Afterwards, we explain the research methods of this study and present the results. Finally, we provide a discussion of our findings and suggest future options for policies and the need for further research.

## Theoretical framework

The concept of circular economy (CE) has been proposed as a promising approach to create more sustainable agri-food systems (Koppelmäki et al. 2021). CE is restorative and regenerative by design, and aims to keep products, components, and materials at their highest utility and value at all times, seeking to ultimately decouple global economic development from finite resource consumption. It serves to replace extract-use-dispose systems with an economic and technological model that is based on principles such as reuse, recycling, reducing and recovering (EMF 2015; Kirchherr et al. 2017). In the context of agri-food systems, it has been proposed that CE includes three stages—food production, food consumption and waste management (Jurgilevich et al. 2016). The food waste hierarchy proposed by Papargyropoulou et al. (2014) applies the CE concept to food waste and serves to inform policy makers on transforming current agri-food systems. This hierarchy comprises the following components, which are ranked from most to least favourable:

1. Prevention;
2. Re-use;
3. Recycling;
4. Recovery;
5. Disposal.

In this study, we put emphasis on the elements of prevention and reuse (1–2).

To analyse how the interrelations between market power imbalances and food loss systematically hamper the development towards a circular agri-food system, we draw on the ‘sociology of markets’ literature. Interestingly, markets as social spaces that are shaped by particular institutions and power relations were traditionally dealt with by only a minority of economists such as Thorsten Veblen, John Commons and Wesley Mitchell (Hodgson 2006, 1998).

Market power from an economic point of view is traditionally defined on the basis of the price setting ability of actors and its effects on economic welfare (Khemani and Shapiro 1993). Industrial organisation literature studies market power and its effects mainly using quantitative approaches. This scientific discipline describes modern agricultural markets as oligopsonies, characterised by increasing concentration, vertical coordination and product differentiation (Russo et al. 2011; Saitone and Sexton 2010; Sexton 2013; Sexton and Xia 2018). Yet, the economic view on market power may not fully capture the complex manifestation of market power and effects beyond market shares, price setting and mark-up (Biely et al. 2019). Fuchs and Clapp (2009) for instance argue that a broader approach to power reveals how it can be employed to influence food system governance patterns and how it enables corporations to shape its constitutive rules and regulations. Devin and Richards (2018) have applied such a power-related approach in the context of food waste to analyse how business organisations can make use of asymmetries to shift responsibilities.

Against this background, this study looks at the institutional preconditions of markets from a sociological point of view by taking the basic considerations of Jens Beckert (2009) as a starting point. Beckert has raised the question of how it is possible that economic activities can be “coordinated” through markets despite the heterogeneous and partly antagonistic motives and interests of their participants. By coordination he means that actors succeed in aligning their actions in ways that allow market exchange to take place. Such coordination is a precondition to the order of markets. Beckert’s (ibid.) point of departure is that markets are highly pre-suppositional arenas of social interaction in which actors are confronted with three fundamental “coordination problems” (ibid.): The problems of (1) cooperation, (2) competition, and (3) value.

1. The cooperation problem arises from the business risks that market actors face because of their incomplete knowledge of the intentions of their exchange partners, the quality of the product they wish to purchase, and incalculable external factors of influence that might hinder the successful order or delivery of the product. The more difficult it is to specify the quality of a product and the less able market actors are to infer each others’ actual intentions, the greater these risks are (ibid.).
2. The problem of competition is related to one of the insights of neoclassical theory that while perfect markets are efficient, in market equilibrium no profit can be made. Suppliers therefore have an interest in establishing market structures that shield them from competitors, which allows them to reduce uncertainty with regard to their profit-making possibilities. Firms alleviate some of the uncertainty created by competition by product differentiation, reciprocal agreements, etc. In sum, however, the structure of competition must be seen as a precarious compromise reflecting the inequalities of the power of actors in the market field (ibid.)
3. The value problem refers to the difficulties of market actors to assess the value of commodities given the multiplicity of goods and their complex quality properties. Only if product qualities and values are distinguishable, will uncertainty be reduced and interest in buying and selling arises. While sellers try to create attachment to their goods on part of buyers through marketing strategies, they must simultaneously react to new and often unpredictable emerging trends. In this sense, the assignments of value are subject to a dynamic process of change and uncertainty and can only temporarily be eliminated for market actors (ibid.).

In this study, we will see that all three coordination problems have a bearing when it comes to understanding the prevalent institutions and practices in fruit and vegetable supply chains in Germany.

## Material and methods

We chose a qualitative research approach, considering that the mechanisms between power imbalances and food loss have not yet intensively been researched. Therefore, in the first place openly addressing the subjective and social constructs of the involved actors is substantial (Flick et al. 2010). In the course of the empirical data collection, we conducted semi-structured expert interviews, which are particularly advisable when processes are complex and not easily accessible (Bogner et al. 2014). This is the case for processes at the producer-retailer interface, in particular with respect to the highly controversial topics of food loss and power imbalances. The approach of a systematising expert interview thereby aims at gathering technical and process knowledge rather than interpretative knowledge (Bogner et al. 2014), which appears to be an adequate form of knowledge with respect to the research questions.

### Acquisition of interview participants

We identified three types of experts as relevant to answer the research questions:

1. Producers (fruit and vegetable growers)
2. Producer organisations of fruit and vegetables, and
3. Food retailers.

In consequence of the heterogeneous structure of the producer-retailer interface of fruit and vegetable supply chains, producer organisations represent only one intermediary within the chain. With 43% of the market volume of fruit (Garming et al. 2018) and 30% of the market volume of vegetables (Strohm et al. 2016) in 2014, a considerable share of German produce is marketed via producer organisations. This study does not consider wholesalers, sorters, packers and storage and logistics providers, due to their declining relevance in most supply chains of fruit and vegetables produced and marketed in Germany (Strohm et al. 2016). As producer organisations have been shown to strengthen farmers bargaining position (Sorrentino et al. 2018; Velázquez and Buffaria 2017), we summarise primary producers and producer organisations as “the production side” or “suppliers”, while retailers are defined as “buyers”. The analysis of power constellations in our case also follows this distinction, although bearing in mind that in some supply chains intermediaries are similarly assumed to exert high levels of market power (Russo et al. 2011).

**Fig. 1:**
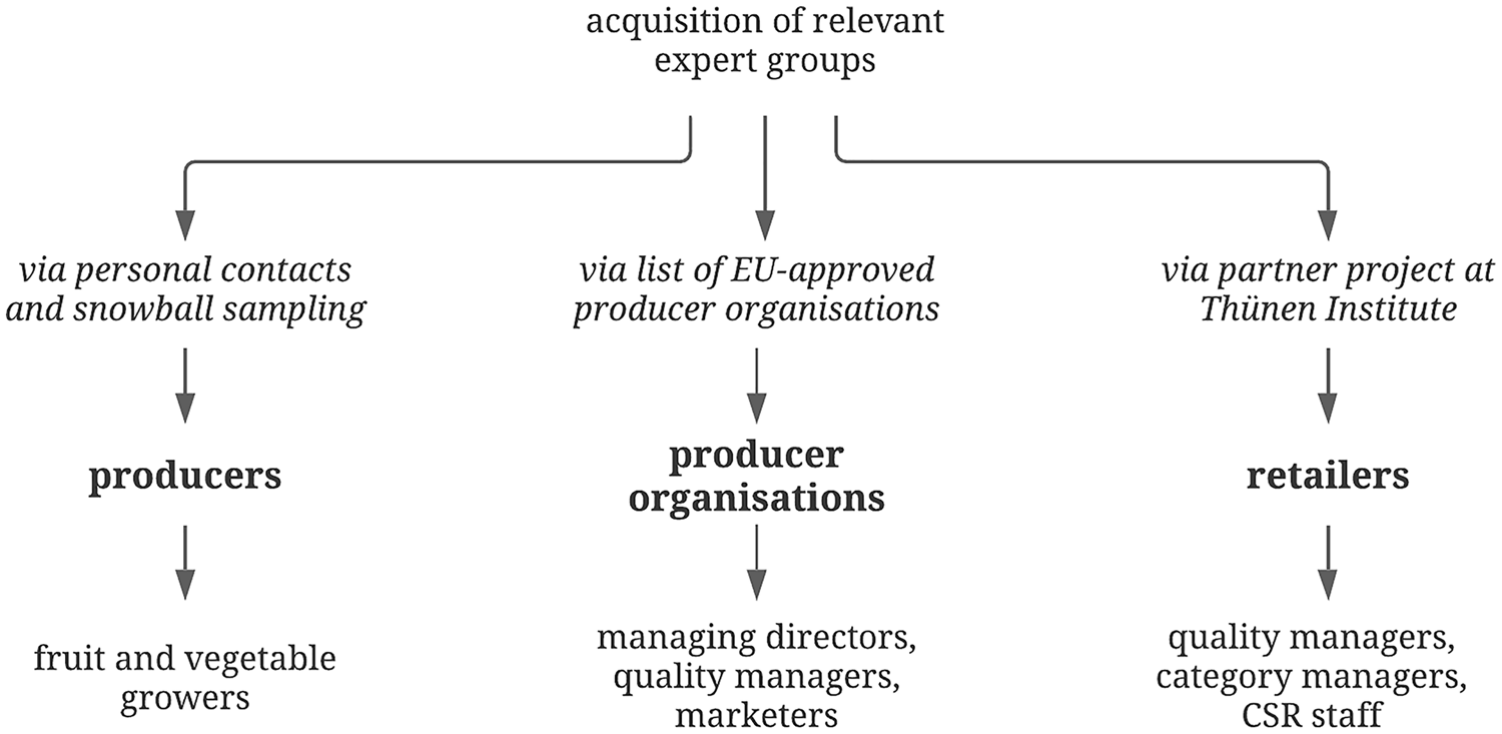
Expert acquisition procedure (means of selection and acquisition, expert group, and position of interviewees within the enterprise)

Experts were acquired by use of personal contacts and snowball sampling techniques, a comprehensive list of EU-approved producer organisations in Germany and a partner project at the Thünen Institute (Fig. 1). The interviewees are active in the fields of fruit and vegetable growing, business management, marketing, quality management, category management and corporate social responsibility (CSR).

### Interview guideline and implementation

We subdivided the interview guideline (S1), developed in accordance with Helfferich (2014) into six main thematic blocks, aimed at gaining insights into the relationship between the different actors of the supply chain with special emphasis on the topic of food loss:

1. Structure of value chain and business relationship
2. Perception of food loss
3. Contracts, agreements, orders, and quantities
4. Quality management and quality standards
5. Trading practices and bargaining power
6. Options for action (policy and private sector)

Overall, we conducted 22 expert interviews with one or two interviewees each between September 2020 and February 2021 with an average length per interview of one hour (Table 1). Seven interviews with primary producers, seven interviews with managers or employees of producer organisations and eight interviews with employees of retailing companies were held. Due to the Covid-19 pandemic, only three interviews could be conducted in person, 15 interviews were carried out via an online video conference tool and four via telephone. Audio files of the interviews were generated and transcribed in accordance with the transcription rules by Dresing and Pehl (2017) followed by a pseudonymisation.

**Table 1:** Characteristics of experts and interviews (region of retailers not shown to preserve anonymity)^a^

Number	Supply chain stage	Date	Region	Produced crops or product range	Type of interview	Length (min)
B12	Primary producer	2020-09-10	Lower Saxony	Carrots and potatoes	In person	45
B16	Primary producer	2021-01-18	Rhineland-Palatinate	Blue berries	Telephone	85
B17	Primary producer	2021-01-20	Baden-Wuerttemberg	Vegetables	Online	38
B18	Primary producer	2021-01-22	Lower Saxony	Blue berries	Online	89
B19	Primary producer	2021-01-22	Baden-Wuerttemberg	Pomaceous fruits	Telephone	59
B20	Primary producer	2021-02-09	North Rhine-Westphalia	Salads and herbs	Online	60
B21	Primary producer	2021-02-10	North Rhine-Westphalia	Vegetables	Online	56
B01	Producer organisation	2020-10-22	Lower Saxony	Onions	In person	61
B10	Producer organisation	2020-11-02	North of Germany	Vegetables	Online	65
B13	Producer organisation	2020-11-03	North of Germany	Vegetables	Online	58
B02	Producer organisation	2020-11-04	Rhenish Hesse	Fruits and asparagus	Online	65
B03	Producer organisation	2020-11-11	Baden-Wuerttemberg	Vegetables	Telephone	49
B04	Producer organisation	2020-11-12	Baden-Wuerttemberg	Pomaceous fruits	Telephone	71
B09	Producer organisation	2020-11-27	North of Germany	Pomaceous fruits	Online	48
B22	Retail	2020-09-16	–	Organic full range	In person	56
B11	Retail	2020-09-22	–	Discounter	Online	44
B08	Retail	2020-11-05	-	Full range	Online	87
B06	Retail	2020-11-09	–	Organic full range	Online	59
B07	Retail	2020-11-09	–	Full range	Online	57
B05	Retail	2020-12-02	–	Discounter	Online	61
B14	Retail	2021-01-06	–	Full range	Online	63
B15	Retail	2021-01-11	–	Organic full range	Online	43

^a^Important cultivation regions, distinct kinds and seasonality of produce, conventional and organic forms of cultivation and a balance between full-range retailers and discounters as well as between large and small companies were considered

### Content analysis

We applied a structuring qualitative content analysis (Kuckartz 2018) with MAXQDA software, which is particularly suitable for analysing technical and process-related knowledge (Bogner et al. 2014). Categories were derived in a hybrid approach combining deductive and inductive logic (Kuckartz 2018). A total of 17 main categories and 29 sub-categories were identified of which ten main categories form the empirical basis of the present study (Table 2). We analysed these categories systematically within segment matrices by theme and per expert group (Kuckartz 2018).

**Table 2:** Excerpt^a^ of the category system developed in content analysis and number of codings

Subordinate category	Codings	Subcategory	Codings
1 General information	23		
2 Relationship between actors	28	2.1 Relationship long-term/on eye level	33
		2.2 Relationship not partner-like/distanced	10
		2.3 Relationship characterised by competition	14
3 Structure of the supply chain	52	3.1 Centralisation/integration	46
		3.2 Supply chain flexibility	45
4 Perception of food loss	31		
5 Orders of retailers	39	5.1 Promotional campaigns	29
6 Quantity estimation and planning	59		
7 Quality standards and specifications	45	7.1 Rejections and complaints	43
		7.2 Packaging specifications	17
		7.3 Pesticide residue limits	20
		7.4 Visual standards/calibre/ripeness	64
		7.5 Legal standards	33
		7.6 Standards set by retailers	43
		7.7 Other standard setters	18
8 Formal contracts	46		
9 Agreements between supply chain actors	58		
10 Trading practices and bargaining power	66		

^a^Only those codes that were considered for this paper and analysed systematically within segment matrices are shown

## Results

**Fig. 2:**
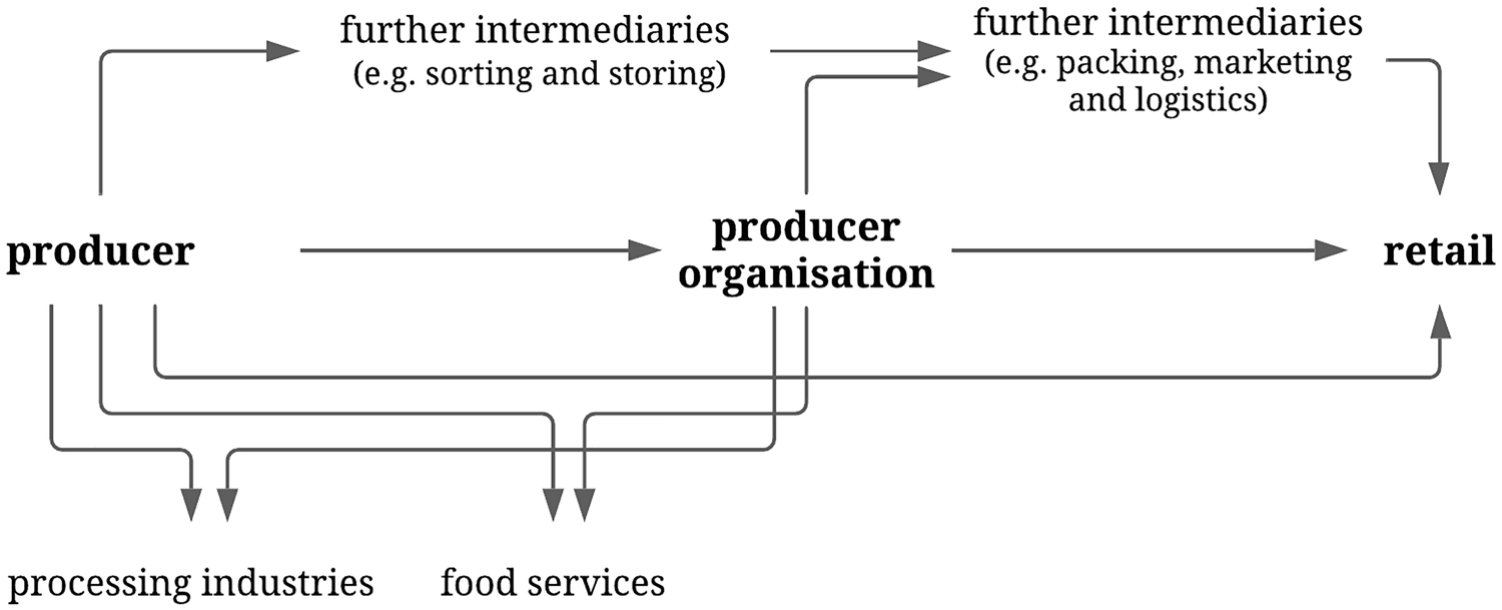
Common structure of supply chains up to retail stage of fruit and vegetables produced and marketed in Germany as depicted by interviewed experts

Supply chains for fresh fruit and vegetables in Germany are structured very heterogeneously and are subject to an ongoing trend of centralisation, concentration and vertical integration, particularly of the retail side (B04:33; B16:9,75–76).^1^ This means that company tasks, such as sourcing and purchasing, are increasingly managed centrally by the firm’s headquarters, as companies are growing in terms of annual sales and number of outlets, while the overall number of competitors is declining. As a result, the upstream supply chain is increasingly coordinated by retailers. Within the interview sample two forms of value chains are included: the direct sale from farmers to retailers and the value chain via one or several intermediaries. For most commodities, fresh fruit and vegetable supply chains are strongly linked to processing industries and food services (Fig. 2).

A broad range of food loss causes was mentioned within the interviews, such as extreme weather events, pests and diseases, logistics and storage problems, false declaration, consumer preferences, etc. However, in this paper we place emphasis on the potential of food loss generation initiated through the patterns of interaction between primary producers, producer organisations and retailers. These patterns rest on particular institutional settings and power relations that we address as inter-stage drivers of food loss and analyse within the following chapters. An overview of these mechanisms exacerbating food loss is presented in Table 3.

**Table 3:** Summary of results concerning materialisation of market power within interactions and corresponding mechanisms that potentially enhance the occurrence of food loss

Chapter	Materialisation of power imbalances	Food loss ﻿provoking mechanism
4.1	Contracts and informal arrangements	Contracts providing no reliability with respect to actually purchased quantity
		Buyers can spontaneously step back from purchase intention
		Exclusive delivery agreements between buyer and supplier impeding from redirecting sales flows
		Lack of short-term informal communication and increasingly detached collaboration
4.2	Quantity estimation and ordering processes	Short-term nature of orders and reorders
		Assignment of delivery obligation by applying auctioning approach
		Inflexible and prematurely fixed promotions not sufficiently buffering harvest peaks
4.3	Product specifications and requirements	Demanding and specific visual and sensory requirements of different retailing companies
		Campaigns with bulky fruit and vegetables not sufficiently coordinated within supply chain
		Individual packaging and pesticide residue limits of different retailers impeding marketing flexibility
4.4	Business relationship and trading practices between production and retail	Occasionally take-back-agreements or short-notice cancellations﻿
		Uncertain nature of orders inducing unpredictability

### Contracts and informal arrangements

The interviews show that formal contracts only set the framework conditions in fresh fruit and vegetable supply chains in Germany. These contracts, also referred to as listing agreements or codes of conduct, lay the foundation of business conduct between retailer and supplier. They for instance contain information on reclamations, duration of listing, obligations, terms of payment, compliance to standards or general product specifications (B02:73; B09:45; B14:96). Contracts generally do not include any delivery specific agreements, such as quantities, prices or purchase commitments. One representative of a producer organisation explains:

“That means, of course, that the framework agreement also regulates the content of the BUSINESS CONDUCT,^2^ it says nothing about the actual business, how much [business] we do together, so it doesn't say ‘we now need 30,000 tons of apples and we will only buy them from you’, such a clause is unfortunately not included” (B09:47).^3^

Written contracts represent the basis of collaboration that informal verbal arrangements build upon when it comes to purchased quantities, e.g., in the wake of annual consultations. Subsequent to these general contracts and informal consultations, retailers place orders in which final purchased quantities are set short-term and in a rather informal manner (Chapter 4.2).

In contrast to most participants, a producer organisation in a special geographic location is assured a purchase guarantee of a certain amount of vegetables already within the contract (B03:23–25). The interviewee sees the producer organisation in a beneficial position compared to others as the supply from the special location is limited and at the same time increasingly in demand (B3:69). Similar to this exceptional case, contracts assuring guaranteed purchase of a predetermined quantity also seem to be common practice in the processing industry (B21:11).

The statements of some experts regarding contracts and arrangements can be linked to the issue of food loss in the early supply chain. Most contracts provide no reliability with respect to the actual purchase of a certain product quantity (B12:35; B20:63). In some cases, the targeted collaboration between supplier and retailer is put into practice. In other cases, the verbally agreed amount is not being met. In consequence of an unforeseeable event, such as weather events, pest infestations or even the Covid-19 pandemic, the retailer is not liable to actually purchase a certain amount of produce. When buyers step back from their purchase intention, this missing liability is a potential cause of loss early in the supply chain. Moreover, a food loss reinforcing situation can arise, when contracts contain clauses preventing producers from supplying third buyers. In this context a blueberry producer describes the contractual terms of a large bundler outside Germany:

“As I said, we had signed a contract with a delivery obligation, and had committed to delivering all of our goods to *wholesaler*^4^ for five years. We would only have the alternative to apply for an exemption, but that would also have to be approved by *wholesaler*. If they didn't approve it, then we couldn't sell” (B18:59).

If the sole supplied buyer does not accept the entire produce due to certain quality specifications or other hindrances, the producer is hardly able to redirect sales flows – a circumstance, which may result in spontaneous food loss at the producer level.

Additionally, all groups of interviewees highlight the importance of short-term informal arrangements regarding food loss prevention. For instance, photos demonstrating product traits and quality are being spontaneously exchanged (B20:23). Retailers can also be informed about unexpected events during production and resulting differences in product qualities or quantities, which may prevent delivery rejections and subsequent food loss (B01:69). A producer organisation, for instance, sells suboptimal product sizes to a packager using these short-term arrangements:

“Well, sometimes there is a customer, who gets a 70/90 or a 70 plus^5^ it’s called sometimes. And then you ask, if it matters if there is something over 90 and if he says, ‘no, it doesn't matter’, then you put the crate in, too. In other words, it only happens when the loading is in progress and the colleague comes over and says ‘there is still a box, can we also load it?’ So it often is very spontaneous” (B01:90).

Particularly those primary producers, who directly deliver seasonal products to retailers, reported a lack of such spontaneous arrangements and indicate a link to food loss. A smaller producer delivering seasonal products to a wholesaler as well as to retailers described the situation:

“And it's a shame that it doesn't meet with understanding. There is absolutely no way I can call my customers, except the wholesaler, who has some room for manoeuvre here […] Others say: ‘No, no, we ordered three pallets, so you have to send the three pallets.’ Yes, that can lead to a refusal of goods. But there is no understanding for my situation [on part of the buyers]” (B16:59).

Moreover, the producer is concerned that central purchasing and the intensified focus of retailers on internal processes, changing staff in the procurement area and an increasing digitalisation of the collaboration might exacerbate the described communication problem and hence boost further food loss (B16:55–61).

### Quantity estimation and ordering processes

The production as well as the retailing side usually carry out an estimation of demanded and supplied fruit and vegetable quantities. Preliminary yield estimation on part of the producers during the flowering period plays a major role for perennial crops, such as stone fruit (B9:51). For annual crops, such as most vegetables, quantities can be adjusted far more flexibly by planting schedules according to the retailers’ demand (B13:46). Retailers mainly estimate their preliminary purchase volumes based on the past years’ demands using prognosis systems (B6:43). However, particularly in smaller retailing companies, the “gut feeling” of procurement staff still seems to play a significant role as the maintenance of prognosis systems can be costly and time consuming (B15:88). Within annual consultations, retailers and suppliers (e.g., producer organisations) usually agree upon approximate purchase volumes over one season, which however only serve as a benchmark. One to two weeks prior to delivery, these quantities are usually fine-tuned and the actual order or retrieval is placed one day before delivery by use of digital systems, e-mail or telephone (B09:53; B16:83). The consulted experts speak of time spans from 12 to 24 h between order and delivery (B13:16), although a longer time span may be stipulated within the terms of delivery (B16:101). Since the predetermined food quantities specified in the annual consultations are based on estimates, it is not until the actual order is placed that the agreement is binding.

Food loss can occur, if the preliminary estimated and actually ordered volumes do not coincide or if estimated quantities are irregularly retrieved. In these cases, initially planned and planted fruit and vegetable quantities eventually cannot be sold and must be tilled or disposed if no other marketing option arises, as a vegetable producer asserts:

“We have a customer who places an order every day for what he needs tomorrow, but he places his order today at 5 or 6 pm, for example, for what I have to deliver at 7 am tomorrow morning. So, I only have a very narrow window to meet the requirements. And if I have the goods ready for harvest, but the orders are suddenly significantly less, then I am not able to sell the entire volume that is in the field” (B20:11).

Due to this time constraint, producers and producer organisations largely rely on their own predictions and practical knowledge and hence pre-pack produce in advance to be equipped for short-term orders and reorders, as the quality manager of a retailing company states:

“I say, it's THE adjustable screw. Because, we pass this adjustable screw on to our suppliers. […] If we place an order today and need something the day after tomorrow, the packing process no longer works. That means they pack and prepare something which they assume will be ordered” (B14:34).

Moreover, the remaining uncertainties regarding eventually ordered and, in some cases, reordered quantities motivate producers to plant more than initially agreed upon, resulting in food loss due to overproduction (B13:47–50). A further food loss driver is the auctioning approach of which some retailers make use. In this case, every one or two weeks, the delivery obligation of a specific product is redefined (B19:68). Producers repeatedly emphasised that the uncertain nature of such an approach can result in food loss, as suppliers can never be fully sure of the possibility to sell their products:

“They jump from one supplier to another from week to week, I've heard that before about *discounter*. […] There are three suppliers offering the product, but *discounter* decides that only one is allowed to deliver this week, while the other two are not. What are the others doing with their product? It still has to be harvested. No, that is clearly not acceptable” (B21:111).

Experts from all interviewed groups confirm that quantity estimations can become even more challenging during promotion periods, when the retrieval of produce becomes more volatile. One interviewee from a retailing company describes:

“We have extremely volatile quantities during promotional activities. Both in one direction and the other. Well, we have advertising, where I need 250,000 raspberries. And then, there is advertising, for which all of a sudden, I only need 100,000 raspberries. That is incredibly difficult for us to estimate” (B08:106).

Accordingly, retailers primarily plan promotions and communicate them to producers or producer organisations mostly two to six weeks before the advertisement period (B09:55). Some experts from the production side depict promotions as becoming increasingly inflexible and prematurely fixed. Hence, they cannot be adjusted spontaneously to harvest peaks. The volatility in orders and the limited flexibility provoke food loss early in the supply chain (B21:55; B20:67).

### Product specifications and requirements

Experts identify product specifications and requirements as another major driver of food loss. These specifications include visual and sensory requirements, such as calibre (size and weight), shape, colouring, taste and the level of ripeness as well as inner qualities such as upper limits for pesticide residues. Not only the product itself, but also its packaging and its production processes can be subject to specific requirements and standards. On the one hand, standards may be set by legal entities in the form of trade category regulations of the EU or criteria set by the United Nations Economic Commission for Europe (UNECE) (B08:75; B10:61). On the other hand, independent and label-based standard defining organisations and companies exist, such as QS, GlobalGAP, IFS, organic farming associations, etc. (B10:21). Furthermore, retailers themselves are indicated as standard setters. While producers and producer organisations claim that retailers’ standards are stricter than legal ones and evoke food loss due to the sorting out of unsuitable produce early in the supply chain (B01:34–35; B09:66–67), retailers generally do not refer to such a correlation (B08:59). All groups of interviewees underpin the importance of raising consumer awareness regarding products that do not meet visual standards. However, producers and members of producer organisations doubt that product requirements arise from customer requests in the first place, but rather from the competitive situation in which retailers find themselves involved. A producer expresses this doubt:

“Today, you have to sort within three millimetres in some cases. I always wonder: ‘Do the retailers even want that?’ […] The consumers can’t even see whether the apple is three millimetres larger or three millimetres smaller” (B19:116).

It was frequently pointed out that visual and sensory specifications set by retailers are rather reliable, well known by all participants of the supply chain and usually not used to artificially reject products at delivery (B20:53). However, some interviewees noticed that requirements become stricter in years of abundant produce and are handled permissively in seasons of short supply (B19:30).

Within the debate on visual requirements, representatives of retailing companies also refer to the marketing of misshapen fruit and vegetables. In this regard, interviewees from the production side see a benefit regarding consumer awareness, although such a practice exists only for selected products (B09:97). However, the potential of selling bulky produce for the reduction of food loss is limited, at least for easily processable fruits and vegetables, as a representative of an apple producer organisation explains:

“[These] apples were already marketed before. Not to retailers, but to processing industries for peeling or juicing. […] In the end you don't get any more money for it, you just get it from someone else" (B02:100–105).

Retailers moreover gave rise to the concern that bulky and over- or undersized products are not readily available in sufficient quantities when asking producers to supply such products (B08:59). In this regard, the production side pointed out that deformed produce is often not even harvested or stored. For the integration of such produce into the supply chain, producers need sufficient assurance that these products will eventually be bought, before adjusting harvesting and sorting processes:

“So, the pickers always work with measurement rings, because we simply do not store cider apples or industrial fruit in the warehouse. Because, I'll put it this way, those often don't cover the storage costs” (B19:20).

Besides visual requirements set by retailers, the interviewed experts underscore two further subjects concerning product requirements: pesticide residue limits and packaging requirements. Although packaging as a protective layer can prevent food loss, it can simultaneously be a driver of loss by reducing marketing options. Packaging, as an integral part of product differentiation, varies considerably between retailers and may frequently be customised (B10:59). Particularly with increasing supply chain integration and products being packed directly after harvest, suppliers are increasingly restricted to a certain marketing channel, as an interview partner from a producer organisation explains:

"In case I have a food tray, for example apples, six apples on a tray with a plastic sheet, then there are usually […] special trays with the logos of the retailers’ own brands, i.e., *full-range retailer*, *full-range retailer*, as they are all called. And I can hardly continue to market them like that. Well, sometimes you would have to repack them” (B04:136).

However, if repacking is too costly, products might rather be disposed of eventually (B14:130).

Similar to packaging, setting individual requirements for pesticide residue limits seems to be common practice of retailers in fruit and vegetable markets in Germany. The interviews suggest that different retailers set individual pesticide residue limits of 100% to 25% of the legally binding maximum value (B20:45; B21:29). Again, the decline in marketing opportunities resulting from these individual pesticide requirements can result in food loss on the part of producers, as an interviewee of an organic retailing company observes:

“Upstream suppliers can only manage this residue requirement in retail if they cultivate the goods specifically for certain commercial channels. […] And the weekly market, which takes the leftovers which no longer come into the food retail for whatever reason, can only absorb to a limited extent” (B14:34).

### Business relationship and trading practices between production and retail

All groups of interviewees use heterogeneous attributes to describe the relationship to other actors of the fruit and vegetable supply chain, ranging from “long-term”, “stable”, “on eye level” and “based on partnership” (B05:47; B6:25) to “acceptable”, “dependent on each other” or even as “imbalanced” (B03:21; B10:87).

The existence of so-called Unfair Trading Practices is denied by most retailers:

“Well, I would say that—well, I can only speak for *own fruit and vegetable agency* for now—we have absolutely no fear or points of contact with so-called Unfair Trading Practices. The things that are on the black list^6^ will be implemented and we are already implementing them today” (B08:138).

In contrast, some representatives of producer organisations and producers have witnessed or heard of practices that they would refer to as unfair. In this context, mainly topics such as terms of payment, payment of promotion costs and price dumping are named and condemned as inacceptable (B16:95). Nonetheless, they generally do not relate this issue to food loss (B13:82). Yet, one interviewee of a producer organisation describes a case in which the costs of unsold products were returned to the producer:

“I think after eight weeks we got the rating^7^ and it was huge and we wondered what was going on and we asked. Well, they packed it and delivered it and then it came back because it was not needed anymore in retail, then it appeared in the rating. Because at that point it was no longer sellable” (B01:183).

However, participants do not refer to return deliveries of unsold products as a systemic problem causing a considerable amount of food loss. Likewise, short-notice cancellations of orders do not appear to happen frequently regarding fruits and vegetables produced and marketed in Germany. In this context, a producer identifies short-term orders as opposed to short-term cancellations of orders as a relevant source of uncertainty, potentially resulting in food loss:

“I might have deliveries of two tons in one day. And the next day zero. Zero. Somehow for me it is of course like a cancellation, but I never got an order” (B16:97).

Interview partners from the production side explicitly identify unequal power relations between the retailing and production side as food loss drivers (B10:87). However, the described mechanisms differ from what the European Commission defines as Unfair Trading Practices. According to the interviews, long-term and balanced business relationships building on a mutual understanding are perceived to effectively prevent food loss along the supply chain.

## Discussion

The discussion is divided into two parts: Firstly, our findings will be reflected on the basis of the theoretical framework. Secondly, these findings will be contextualised and compared given insights from other countries with a specific focus on the issue of Unfair Trading Practices and power imbalances.

## Food loss from a market sociology perspective

Based on the insights of Jens Beckert (2009) we suggest that market interactions need to be coordinated. By coordination Beckert (ibid.) means that actors need to reduce the fundamental uncertainty inherent in market relations in regard to (1) their incomplete knowledge of the intentions of their exchange partners (cooperation problem), (2) their personal profit expectations (competition problem), and (3) the difficulties of assessing and fixing the value of commodities (value problem) before the exchange of goods can take place. As will be shown, all three mentioned coordination problems have a bearing in current fruit and vegetable supply chains in Germany and help to identify inter-stage drivers of food loss and the interrelation between market power, food loss and waste, and economic loss.

1. The interviews have shown that formal contracts set only the framework conditions for market exchange and form the basis of collaboration. Informal arrangements then serve to place actual short-term orders of specified quantities. Thus, in the supply chains studied, the cooperation problem is solved via a combination of formal and informal modes of governance that are also an expression of underlying power relations. From the producer perspective, most contracts do not provide any reliability with respect to the actual purchase of specified amounts of produce. This lacking liability can cause material and financial loss on the part of producers when retailers step back from their purchase intention—especially when producers are bound by contract clauses to sell their produce to only one defined buyer.
2. The problem of competition becomes important when it comes to quantity estimation and the forecast of demand. Since retailers constantly need to highlight their recognition value in a highly competitive environment, they need to offer their customers the broadest possible variety of high-quality products (Hooge et al. 2018). In this context, retailers estimate the purchase volumes of the next year on the basis of past years’ experiences. To be able to source fresh produce on a regular basis and to adapt to short-term changes in demand, retailers make use of short-term orders to avoid economically harmful stock-out (Avlijas et al. 2015). Producers have developed coping strategies such as to pre-pack produce in advance to be equipped for short-term orders or reorders. In case own preparations do not fit with retailers’ orders, again, material and financial loss appear while producers have to bear the costs.

- As the interviews show, the described problem of producers to estimate demanded quantities becomes especially difficult in times when retailers run promotion campaigns. As these campaigns are directed against competitors to attract customers and to raise profits, they are seldom communicated to the producers more than six weeks in advance, nor are they adjusted flexibly enough to meet harvest peaks. The unpredictability in combination with the mere size of ordered quantities during promotion periods can result in producers tilling existing crops, if eventually ordered quantities and produce ready for harvest do not coincide. This again can result in material and financial loss to the detriment of the producers.

3. Last but not least, also the value problem can be consulted to explain a food loss fraction that occurs due to quality requirements. This is caused by the fact that the value of a product is nowadays defined by a broad range of specifications laid out in legal standards, independent and label-based standards as well as private standards by retailers. The variety of requirements concerning pesticide loads and packaging by distinct retailers forces producers to either specialise on particular marketing tracks or to fulfil the maximum requirements in the market. As a consequence, producers either have to follow an “all eggs in one basket” strategy or increase production costs to meet the highest standards. None of these strategies goes without the risk of decreasing margins. Apart from that, it is noteworthy that even the sale of deformed produce does not necessarily come without extra costs on part of the producer, since an integration of such produce into the supply chain would involve costs to adjust related harvesting and sorting processes. In this context, the question arises of who bears the costs, if not the producers. From their perspective, however, it seems odd to invest in a production process optimisation to sell their produce at a rate which is not necessarily higher than for regular produce.

In sum, we show that the generation of food loss in current supply chains of fruit and vegetables can arise due to the specific institutional ordering of markets, which are an expression of power relations. Thus, if the aim is to avoid the production of food loss, there is scope to not only focus on technical solutions, but also to transform prevalent market structures and create incentives, policy instruments and alternative marketing options to empower producers and producer organisations to be able to solve their specific coordination problems by negotiating with retailers at eye level. The preceding integration of food loss provoking mechanisms into the theory of coordination problems shows that the question of risk bearing is crucial to understand where food loss is triggered and where actual loss and its costs occur (Gillman et al. 2019).

### Food loss from a comparative, policy-related perspective

The findings of this paper suggest that market power imbalances play a pivotal role in the depicted supply chain interactions inducing food loss. However, the mechanisms through which market power imbalances and risk shifting behaviour result in food loss diverge from the expectations based on the literature and the recent EU directive (European Parliament 2019). Piras et al. (2018), Sinclair Taylor et al. (2019) and Feedback (2017) give rise to the assumption that UTPs represent major drivers of food loss and waste along the supply chain. Accordingly, short-notice cancellations or order changes as well as the artificial reduction of initially ordered quantities by use of inconsistently applied quality criteria are causing major food loss. For the UK, Rakesh and Belavina (2020) describe that the sponatneous alteration of quality requirements is sometimes used as a means to return no longer required produce, a situation previously found by Eriksson et al.(2017), Devin and Richards (2018) and Feedback (2017) as well. The finding that retailers use standards regarding visual and sensory traits, (Beausang et al. 2017; Porter et al. 2018; Richards and Hamilton 2020), pesticide residue limits (Ludwig-Ohm et al. 2019; Meyer et al. 2017), and client-specific packaging (Meyer et al. 2017) to govern the supply chain beyond their own organisation (Devin and Richards 2018; Fulponi 2006), can be supported by the results of this paper. However, an intentionally inconsistent application of quality requirements by retailers to justify rejections could not be found. Similarly, short-term order cancellations, sending back or charging the cost of unsold products in the form of take-back-agreements (Eriksson et al. 2017; Ghosh and Eriksson 2019; Gille 2013) or backward selling contracts (Rakesh and Belavina 2020) were not identified as a systematic problem for fruit and vegetables cultivated and supplied in Germany. In this case, a system is running which makes such practices unnecessary. Due to low liability regarding quantities, missing purchase commitment, and short-term orders and reorders instead of short-term cancellations, the production side of the value chain is burdened with the consequences of potential risks and food loss. In this sense, the practices of take-back-agreements and short-notice cancellations described within the directive on Unfair Trading Practices (European Parliament 2019) are not sufficiently addressing the core problem in this case. As the quantitative assessment of food loss and waste prevention actions is crucial (Goossens et al. 2019), it should be observed whether an imposition of more fixed terms through regulation will actually reduce overall food loss and waste. It might on the other hand reduce flexibility to cope with unexpected changes and thus provoke even more environmentally harmful food loss and waste down the supply chain (Gillman et al. 2019). The horizontal integration of farmers in producer organisations (Porter et al. 2018; Velázquez and Buffaria 2017) as well as the diversification of their distribution channels (Chaboud and Moustier 2021; Devin and Richards 2018) and a reduction of excessive product differentiation and specification (Ludwig-Ohm et al. 2019; Thies et al. 2021) might be more effective mechanisms to enhance producers’ bargaining position and counteract food loss.

All in all, to create less waste in more sustainable fruit and vegetable supply chains, it must be recognized that food loss can be the outcome of rational decisions by market actors in consideration of their costs and particularly also risks (Golan et al. 2019; Kuchler and Minor 2019; Rutten 2013). The topic of power imbalances and its arising risk and incentive allocation must thus be considered further. A more balanced risk-sharing along the supply chain may force all actors to optimize activities and prevent a food loss fraction out of economic considerations (Koester 2014). This would be also favourable from a CE and food waste hierarchy (Papargyropoulou et al. 2014) point of view. It should not be neglected that preventing the food loss fraction arising from inter-stage drivers of food loss may incur costs and risk on the part of buyers. Therefore, it must be questioned whether cooperative policy approaches such as voluntary agreements (Burgos et al. 2019) alone will suffice in this particular case or whether further instruments will be required (Garske et al. 2020).

## Conclusions

To conclude, inter-stage drivers of food loss play a pivotal role in the context of fruit and vegetable loss in Germany. In this context, powerful retailers use their position to solve the uncertainties arising from ‘cooperation problems’ within markets to a large extent at the expense of producers. Underlying mechanisms are based on specific institutional frameworks, which vary between countries, products and supply chains. In the case of fruit and vegetables cultivated and supplied in Germany, we have identified the following key inter-stage drivers of food loss:

1. Low liability regarding quantities,
2. Short-term orders and reorders,
3. Missing purchase commitment,
4. Client-specific requirements on appearance, packaging and pesticide residue limits
5. Top-down implementation of orders, promotions and product specification.

We argue that policies restricted to voluntary actions at individual stages of the food supply chain may be insufficient to tackle this particular food loss fraction as the incentive for retailers to shoulder costs and risks resulting in upstream food loss prevention is low. To develop purposeful policy instruments targeting these inter-stage food loss drivers, we suggest for politics and future research to put emphasis on how to:

1. Create more liability within market transactions;
2. Adjust and unify product specifications;
3. Propagate a bearing of costs of process and specification adjustments shared by producers and retailers;
4. Design more flexible promotional campaigns harmonised with producer capacities;
5. Maintain informal modes of governance within supply chains despite further concentration, centralisation and digitalisation; and
6. Limit structural power imbalances and risk bearing in contemporary fruit and vegetable supply chains, e.g. through fostering horizontal integration and alternative marketing channels.

Further research is moreover required on the empirical evidence and quantification of the effects of UTPs in general and with a specific focus on imported products that cannot be ordered just-in-time. A further quantitative evaluation of the effects of food loss drivers identified within this paper, as well as the evaluation of counteracting measures, would be a desirable next step in research. In this context, measures to balance power between producers and retailers would also have to be analysed in consideration of potential rebound effects and should not create new inflexibility or simply shift food loss down the supply chain. We argue that a deeper understanding of the interrelationship of cooperation problems in markets will be helpful to identify and to uncover different facets of power imbalances and the shifting of business risks in food markets. Such an understanding is necessary to refine the current debate on creating CEs and sustainable food systems, which is too often coined by the question on mere technical feasibility, rather than systemically impeding institutions and practices.

## Supplementary Information

Below is the link to the electronic supplementary material.

Supplementary file1 (PDF 279 KB)

## Footnotes

1 Statements of the results section that are not underlined by a direct quote, are supported by indicating exemplary interview passage(s).

2 Capital letters in quotes indicate loud and accentuated pronunciation.

3 Quotes used within the results section were translated by an English native speaker and grammatical and linguistic errors were corrected to improve understanding and reading flow.

4 Parts of quotes written in italic were pseudonymised.

5 Calibre category of onions (diameter in millimetres).

6 List of “Unfair Trading Practices” within the directive of the European Commission that must be banned within EU member states, as opposed to “grey list” including practices that may persist if explicitly agreed upon by the involved supply chain actors.

7 Monetary discount, e.g., due to product shares not fulfilling the required quality.
