# Supplementary material for: Market power and food loss at the producer-retailer interface of fruit and vegetable supply chains in Germany
Source: Sustain Sci. 2022 Jan 15;17(6):2253–67. doi: 10.1007/s11625-021-01083-x (PMC8760580; doi:10.1007/s11625-021-01083-x)
Supplement: Supplementary file 1 — Supplementary file1 (PDF 279 KB) [file 11625_2021_1083_MOESM1_ESM.pdf]

Supplementary Material (S1): Guideline for expert interviews on market power and food loss of fruits and vegetables at the producer-retailer interface

Introduction and Warm-Up

Please tell me a little bit about yourself:
How did you come to the [retailing company/producer organization/farm business]?
What exactly are your tasks at [retailing company/producer organization/farm business]?

Thematic block 1: Structure and business relationship between production and retail

Key question / stimulus / invitation to explain		
**1.1) If you are thinking about the agricultural value chain of [fruit/vegetables] from horticulture/primary production to retail: What steps and actors lie between production and point of sale?**		
**Content aspects of question**	**Maintaining conversation**	**Possible inquiries**
• Overview of supply chain and commodity flow<br>• Important actors in the field<br>• Structure of food and vegetable sectors	• What happens within this step?<br>• Could you provide more details on this step?<br>• What happens before/ afterwards?	• How many buyers do you supply/ from how many suppliers do you source produce?<br>• Which actors are you dealing with in everyday business?

Key question / stimulus / invitation to explain		
**1.2) Please describe your business relationships with producers and suppliers from whom you source your fruit and vegetables/ with retailers to whom you are selling your fruit and vegetables.**		
**Content aspects of question**	**Maintaining conversation**	**Possible inquiries**
• Profound insights into the interface<br>• Perception of business relationships<br>• Potential reference to bargaining power of actors	• What kind of producer/ organization/ company is this exactly?	• Which further marketing channels/ suppliers exist?<br>• What is your personal impression of this business relationship (in comparison to others)?

Thematic block 2: Perception of main drivers of food loss

Content-related input of interviewer: "In the course of the interview I will use the term 'food loss between primary production and retail'. By this I mean all kinds of food intended for human consumption but eventually not used for this purpose. Included here is also food which is used in alternative ways such as feed, biogas, fertilizer, etc. In this case, we are talking about food lost up to the retailer's 'doorstep' or warehouse, including pre-harvest and harvest losses. This does not include food products that are disposed from the retailers' shelves. [Show Figure 1 to interviewee]"

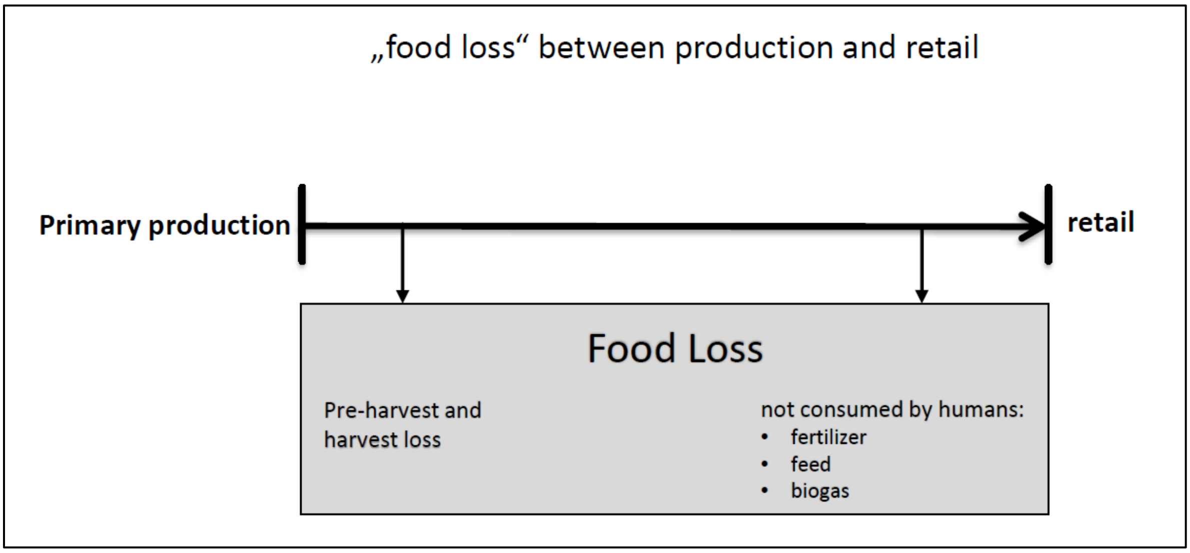

Fig. 1 Illustration of "food loss" definition in the course of the interview

**Key question / stimulus / invitation to explain**		
**2.1) In your view, to what extent does food loss between primary production (including pre-harvest) and retail occur, so that food products cannot, as initially intended, be utilized for human consumption?**		
**Content aspects of question**	**Maintaining conversation**	**Possible inquiries**
• General awareness of the interviewee of food loss<br>• Perception of scale and relevance of food loss	• What about other stages/ products?	• At which point of the supply chain/ for which products?<br>• How much loss roughly occurs where?<br>• Does this include pre-harvest losses?

Key question / stimulus / invitation to explain		
**2.2) What are, from your perspective, the main drivers of food loss of fruit and vegetables between primary production and retail?**		
**Content aspects of question**	**Maintaining conversation**	**Possible inquiries**
• Perception of food loss drivers and their relevance<br>• Perception of actors' own role and role of others in the context of food loss	• How does this result in food loss?<br>• Could you think of further drivers?	• Who is responsible for this?<br>• How could you influence this driver and to what extent are you doing this already?

Thematic block 3: Contracts, agreements, orders and quantities

Key question / stimulus / invitation to explain		
**3.1) Please describe how contracts and arrangements on deliveries of fruit and vegetables with producers or suppliers/retailers come about and how they are designed?**		
**Content aspects of question**	**Maintaining conversation**	**Possible inquiries**
• general insight into contracts and arrangements<br>• hints to trading practices and bargaining power<br>• potential food loss drivers within contracts/arrangements	• Could you provide some more details on this?	• What is the usual time period of these contracts/ arrangements?<br>• What is the content of these contracts?<br>• How have contracts and arrangements developed over the years?

Key question / stimulus / invitation to explain		
**3.2) Please describe how the quantity estimation of a fruit/vegetable order is carried out and how it is communicated to producers and suppliers. If you wish, you can do so by means of an example.**		
**Content aspects of question**	**Maintaining conversation**	**Possible inquiries**
• existence of demand prognosis<br>• problems in ordering and quantity estimation as food loss drivers<br>• bargaining power and shouldering of economic risk	• How exactly is this carried out?<br>• Could you explain this procedure in more detail?	• Which criteria are applied regarding the volume of an order?<br>• How far in advance is the decision on the delivery volume made?<br>• Under which circumstances may the delivery volume be adjusted afterwards?

Key question / stimulus / invitation to explain		
**3.3) In what sense may contractual terms or the management of orders and quantity estimations positively or negatively affect the occurrence of food loss?**		
**Content aspects of question**	**Maintaining conversation**	**Possible inquiries**
• Contractual clauses and other mechanisms resulting in food loss<br>• Shifting of economic risk through contract clauses and other mechanisms	• What other stipulations are included?<br>• Could you name any specific clauses?	• How does this promote/ impede food loss?<br>• Could you provide a practical example?<br>• Do you perceive a general correlation or is this an individual case?

Thematic block 4: Quality management, quality standards and return deliveries

**Key question / stimulus / invitation to explain**		
**4.1) Please describe the quality management process along the supply chain. Which criteria influence the decisions made to actually offer products for sale?**		
**Content aspects of question**	**Maintaining conversation**	**Possible inquiries**
• Details on quality standards, particularly of retailers<br>• Strictness and flexibility of quality standards as potential food loss drivers<br>• Perception of "private" quality standards by actors	• How exactly is this control carried out?<br>• What happens afterwards?	• At which point does the product change the owner?<br>• Are these criteria communicated in advance or applied flexibly?<br>• Who is monitoring when if products adhere to the standards?<br>• How would you describe retailers' standards as compared to official ones?

**Key question / stimulus / invitation to explain**		
**4.2) What happens if products do not meet the required quality standards?**		
**Content aspects of question**	**Maintaining conversation**	**Possible inquiries**
• Existence of return deliveries<br>• Reasons for return deliveries<br>• Economic risk of rejection and return deliveries	• What other options exist?<br>• How exactly is this carried out?	• Under which circumstances may rejections occur?<br>• In what way are return deliveries stated within contracts?<br>• Who carries the costs?

Thematic block 5: Trading practices and bargaining power

**Key question / stimulus / invitation to explain**		
**5.1) The topic of trading practices between production and retail is repeatedly in the focus of public interest and also being discussed in the context of food loss. To what extent are you aware of the debate and what is your personal opinion on it?**		
**Content aspects of question**	**Maintaining conversation**	**Possible inquiries**
• Awareness of directive on unfair trading practices<br>• Perception of fairness within supply chain<br>• Perception of correlation between trading practices and food loss	• What do you think about this practice?	• How would you describe trading practices between production and retail?<br>• Which practices could, in your view, trigger food loss?

**Key question / stimulus / invitation to explain**		
**5.2) Within the debate on trading practices there is talk of *take-back-agreements* and *short-notice cancellations*, or more specifically sending back unsold products to the producer and short-term cancellation of deliveries, respectively. Would you please tell me about your experiences in this context, if you are aware of these practices from any context?**		
**Content aspects of question**	**Maintaining conversation**	**Possible inquiries**
• Correlation of "unfair" trading practices and food loss<br>• Concrete practices as food loss drivers	• Could you share experiences from another context?	• When exactly/for which products does this take place?<br>• How exactly are such arrangements designed?<br>• What other practices could result in food loss?

Thematic Block 6: (Policy) options for action to reduce food loss

Key question / stimulus / invitation to explain		
**6.1) What change is needed to reduce food loss within the supply chain between primary production and retail?**		
**Content aspects of question**	**Maintaining conversation**	**Possible inquiries**
• Perception of individual and policy options for action<br>• Perception of self-efficacy	• What else would have to change?	• Who is responsible for such a change?<br>• What could politics do?<br>• What else could you as a firm bring about?

Key question / stimulus / invitation to explain		
**6.2) Which support and action do you expect or require from politics to reduce food loss between primary production and retail?**		
**Content aspects of question**	**Maintaining conversation**	**Possible inquiries**
• Alternative policy approaches<br>• Requirement for intervention<br>• Potential regulative, market-based and cooperative policy instruments	• Could you think of another instrument?	• Why would this be advisable?<br>• Where exactly would this intervention be required?

Interview Conclusion

Is there anything you would like to add or a topic that we have missed in the course of the interview and you would like to address?
